# Germinal ovarian tumors in reproductive age women: Fertility-sparing and outcome

**DOI:** 10.1097/MD.0000000000022146

**Published:** 2020-09-25

**Authors:** Miriam Dellino, Erica Silvestris, Vera Loizzi, Angelo Paradiso, Rosalia Loiacono, Carla Minoia, Antonella Daniele, Gennaro Cormio

**Affiliations:** a Gynecologic Oncology Unit, IRCCS Istituto Tumori “Giovanni Paolo II”, Bari, Italy; b Department of Biomedical Sciences and Human Oncology, Unit of Obstetrics and Gynaecology; c IRCCS Istituto Tumori “Giovanni Paolo II”, Bari, Italy; d Haematology Unit, IRCCS Istituto Tumori “Giovanni Paolo II”, Bari, Italy; e Biology Research, Department of Experimental Oncology, IRCCS Istituto Tumori “Giovanni Paolo II”, Bari, Italy.

**Keywords:** fertility-sparing surgery, malignant ovarian germ cell tumors, outcome, ovarian neoplasms, reproductive age

## Abstract

MOGCTs (malignant ovarian germ cell tumors) are rare tumors that mainly affect patients of reproductive age. The aim of this study was to evaluate the fertility and survival outcomes in young women with MOCGTs treated with fertility-sparing surgery (FSS).

From 2000 to 2018, data from 28 patients of reproductive age with a diagnosis of MOGCT at the University of Bari were collected. Most received FSS, and in patients treated conservatively, the reproductive outcome and survival were investigated. Data of patient demographics, clinical presentation, oncology marker dosage, staging, type of surgery, histological examination, survival, and reproductive outcome were collected from hospital and office charts. All informed consent was obtained from all patients. The median age was 24 (range: 9–45 years). The majority of the patients had stage IIIC. Twenty-four woman received FSS consisting of unilateral ovariectomy and omentectomy, whereas only 4 women, based on their stage (IIIC), received a radical surgery (hysterectomy with bilateral adnexectomy, lymphadenectomy, and omentectomy). Our study shows that FSS in MOGCTs can produce good results both on reproductive outcomes and on survival. Indeed, in our group, there was only 1 case of exitus as result of recurrence. Furthermore, patients after FSS maintained normal ovarian function and 5 of 5 women who tried to get pregnant succeeded spontaneously. The median follow-up was 90 months (range 3–159).

Conservative surgery for MOGCTs should be considered for women of reproductive age who wish to preserve fertility.

## 1 Introduction

Ovarian germ cell tumors (OGCTs) represent 20% to 25% of all ovarian neoplasms, whereas malignant ovarian germ cell tumors (MOGCTs) comprise only 5%.^[1,2]^ MOGCTs show a peak prevalence in young women and adolescents. Approximately 60% of ovarian tumors are germ cell tumors in patients younger than 20 years, and one-third of these cases are malignant.^[3]^ MOGCTs include several histotypes, all deriving from primordial germ cells of the ovary, and represent a heterogeneous group of tumors with variable biological behavior, clinical presentation and prognosis.^[4]^ There is a variety of histologic types: dysgerminoma, immature teratoma, endodermal sinus tumor, choriocarcinoma, polyembryoma, and mixed MOGCTs.^[5]^ These tumors differ from epithelial ovarian cancer (EOC) by incidence at an earlier age, for their almost unilateral localization (95% of cases), for their high rate of growth, a rare tendency to spread, and finally for their good prognosis.^[6,7]^ This is explained by the numerous diagnoses at early stages and their high chemosensitivity.^[8]^ Therefore, the treatment of this pathology in early stages requires only surgery, while in the presence of high-risk factors or advanced disease, after surgical treatment, chemotherapy is highly recommended.^[9,10]^ The most-used chemotherapeutic protocol is the combination of Bleomycin, Etoposide, and Cisplatin (BEP) or Bleomycin, Vinblastine, Cisplatin (PVB) for 4 to 6 cycles, and this is based on histotype and stage.^[11]^ Patients are followed up with abdominal-pelvic examination and ultrasound, complete blood count, and a biochemistry profile every 2 to 3 months for the first 2 years, at semi-annual intervals up to year 5, and annual intervals thereafter. Chest X-ray is ordered at annual intervals or in case of clinical suspicion. Computed tomography of the thorax and abdomen is performed every 6 months for the first 2 years, and at annual intervals up to year 5.^[12]^ A variable long-term survival rate of 82% to 100% is reported in the literature in the early stages and 75% in the advanced stages.^[12]^

Considering the frequent incidence of MOGCTs in young women and the high survival rate, clinicians should consider fertility-sparing surgery (FSS).^[13]^ Correct information about the risks of iatrogenic infertility and the strategies available to reduce the incidence of this effect (reproductive counseling) should be offered to young cancer patients immediately after diagnosis, at subsequent stages of the disease, and before the start of treatment.^[14]^ The main techniques of fertility preservation in patients who need to undergo cancer treatments are cryopreservation of embryos or eggs, cryopreservation of ovarian tissue, gonadic suppression with similar gonadotropin-releasing hormone (GnRH) analogues, and conservative surgery. FSS is identified as the cornerstone of early-stage MOGCT treatment. On the contrary, for the advanced stages, the possibility of performing an FSS in young patients who desire a pregnancy should be assessed in a personalized manner and after counseling with the patient.^[10]^ Indeed, current literature presents reassuring data relating to favorable overall survival (OS) and reproductive outcome after FSS.^[11]^ In this study, we analyzed survival outcomes in women of childbearing age diagnosed with MOGCTs and treated with FSS. We also evaluated changes in the menstrual cycle and post-treatment pregnancies.

## 2 Material and methods

From 2000 to 2018, data from all women of reproductive age with a diagnosis of MOCGTs at the University of Bari were collected. Data of patient demographics, clinical presentation, oncology marker dosage, staging, type of surgery, histological examination, survival, and reproductive outcome were collected from hospital and office charts. Each patient was given a descriptive form of the study and was formally invited to participate. After having agreed, informed consent was submitted to all patients. All procedures performed in this study were in accordance with the Helsinki Declaration as revised in 2013. In addition, patients were also informed that the data collected for this study are protected by the Privacy Act; therefore, they were collected and used after obtaining written authorization from each patient for the use of personal data for scientific purposes only. The evaluation of the disease stage was performed using the Federation of Gynecology and Obstetrics (FIGO) classification,^[14]^ whereas the histopathological definition was evaluated according to the classification of germ cell tumors of the ovary of the World Health Organization (WHO).^[15]^ A cytological analysis of the ascitic fluid or the peritoneal washing and an intraoperative histological examination was performed in all cases. In addition, all patients presented an extemporaneous germ cell tumor subsequently confirmed at the definitive histological examination. The “maximum” debulking was defined as a tumor residue = 0 after primary or recurrent surgery, “optimal” in the case of a 1 cm tumor residue, and “not optimal” >1 cm. Disease-free survival (DFS) was defined as the period between diagnosis and recurrence, whereas OS was identified as the period between diagnosis and the time of death or last follow up.^[16]^ Adjuvant BEP or PVB (average of 4 cycles every 3 weeks) was indicated for patients with IC or higher disease, immature teratoma or high-grade tumors, and residual disease after cytoreductive surgery. Patients were followed up with abdominal and pelvic examination and ultrasound, blood count, and tumor marker dosage every 2 to 3 months for the first 2 years, at semi-annual intervals up to year 5, and at annual intervals thereafter. Computed tomography of the thorax and abdomen was performed every year for the first 5 years. Chest X-ray was indicated at annual intervals for the first 2 years. The limitations of this study are represented by the limited number of cases, and the retrospective analysis.

## 3 Results

Twenty-eight patients with a diagnosis of MOGCTs were studied. The median age was 24 (range: 9–45 years). The majority of the patients had stage IIIC. Most received FSS consisting of unilateral ovariectomy and omentectomy, whereas only 4 women, based on their stage (IIIC), received a radical surgery (hysterectomy with bilateral adnexectomy, lymphadenectomy, and omentectomy). The median follow-up was 90 months (range 3–159) (Table 1). The onset of the disease was pain in 18 (64%), abdominal distension in 26 (26%), ascites in 2 (7%), dyspareunia in 3 (10%), weight gain 2 (7%), vaginal bleeding 1 (3.5%), and symptomless in 3 women (10%). The average diameter of the neoplasms was 9.2 cm (range 5–20). An increase was also recorded in the following tumor markers: carbohydrate antigen (CA) -125 in 3 (10.7%), beta-human chorionic gonadotropin (β-HCG) in 6 (21.4%), alpha-fetoprotein (AFP) in 11 (39%), CA 19.9 in 2 (7%), and CA-15.3 in 1 patient (3%). None of our patients showed an increase in carcinoembryonic-antigen (CEA). Tumor histology included 4 (14%) teratomas, 11 (39%) dysgerminomas, 3 (10%) endodermal sinus tumors, and 10 (35%) mixed MOCGTs tumors. Ten women had I A (35%), 5 presented stage I C (32%), 5 stage II A (18%), and 4 (14%) stage IIIC disease. Therefore, a total of 18 patients were treated with adjuvant therapy: 16 with BEP and 2 with PVC. The chemotherapeutic regimen was administered for 4 cycles every 3 weeks (Table 1). Three women (12%) treated with FSS with a histology different from dysgerminoma had a recurrence 6 years after treatment; therefore, a completion of surgery was performed. Of the 28 women, 2 women did not survive: 1 with a diagnosis of endodermal sinus tumor died due to disease progression despite radical surgery and chemotherapy, and another died due to severe fatal toxicity after the first cycle of PVB chemotherapy. With a median follow-up period of 90 months, 5-year OS rate was 85% and the record of recurrence rate was 12% with a DFS of 88% in our population. Moreover, fertility preservation techniques were used in 8 patients by performing GnRH analogues during chemotherapy, and in 2 cases through cryopreservation of ovarian tissue and cryopreservation of oocytes upon commencement of treatment, respectively. In these last 2 cases, both patients later had spontaneous pregnancies without having to resort to thawing. Of the 28 patients of child-bearing age diagnosed with MOCGTs, excluding the 4 patients who had a hysterectomy after diagnosis, 3 after recurrence, 1 patient with pure gonadal dysgenesis (Swyer syndrome -karyotype 46 XY), and 1 death after chemotoxicity, we investigated the regularity of the post-treatment menstrual cycle and reproductive status. Of 19 women, 15 (78%) reported regular menstrual cycles during and after chemotherapy; on the contrary, the remaining 4 (21%) presented amenorrhea during chemotherapy but reported regular cycles after the end of treatment. Regarding the reproductive outcome, 4 patients had a pregnancy before the disease without attempting further conception, 11 patients (average age 32 years) declared that they were not currently interested in childbearing, 1 women had a spontaneous abortion (SAB) at 7 weeks, 1 had a voluntary pregnancy interruption (IVP) at 22 weeks following a diagnosis of multiple fetal malformations, and the remaining 5 reported having achieved pregnancy through spontaneous conception and with vaginal delivery. Of these 5 women, the median time to achieve a pregnancy was 8 years from surgery (range 3–15 years) and 4 of them had a combination of BEP + GnRH analogues, whereas 1 only chemotherapy after surgery (Table 2).

**Table 1:**
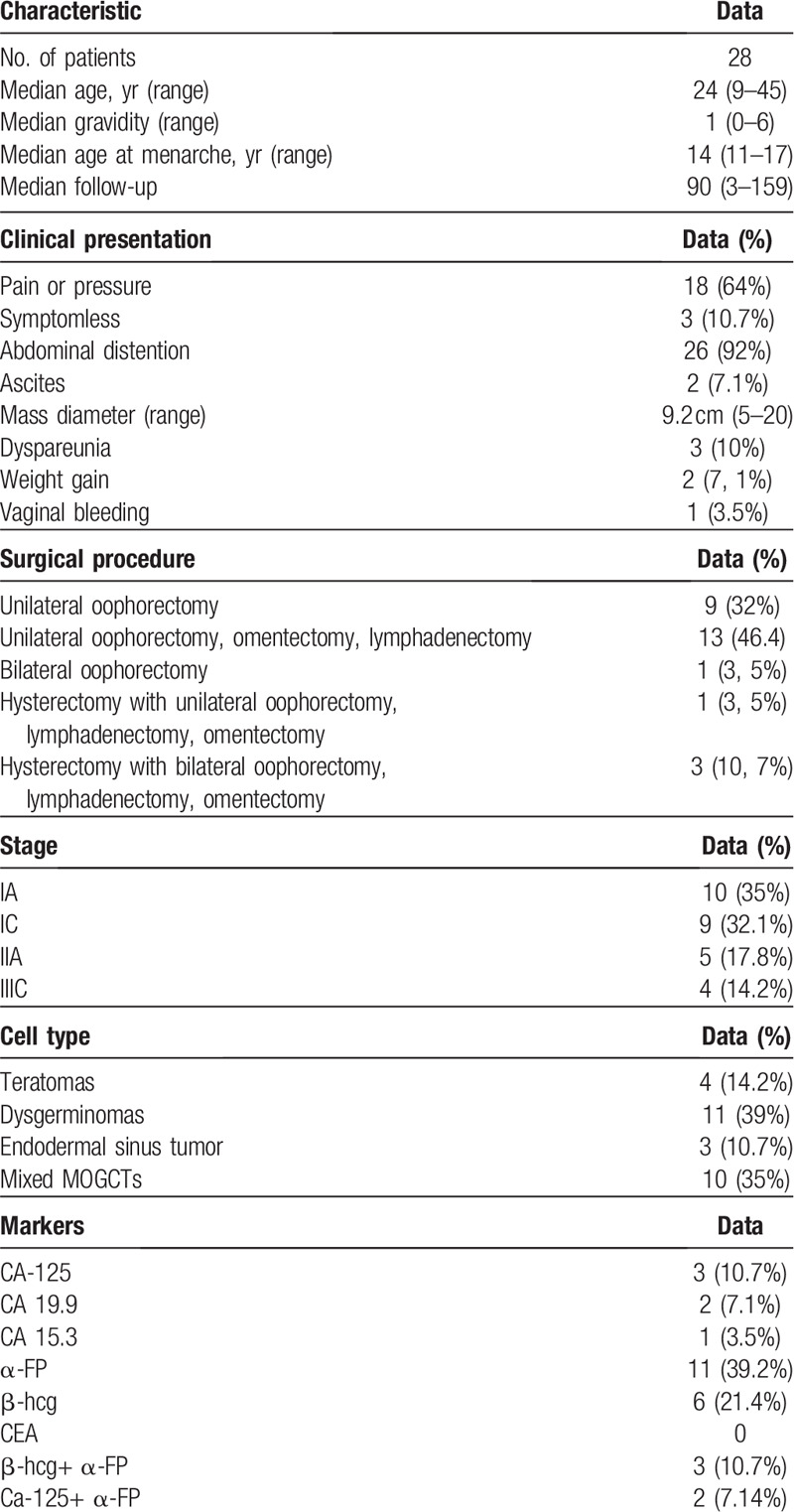
Patient characteristics and treatment data.

**Table 2:**
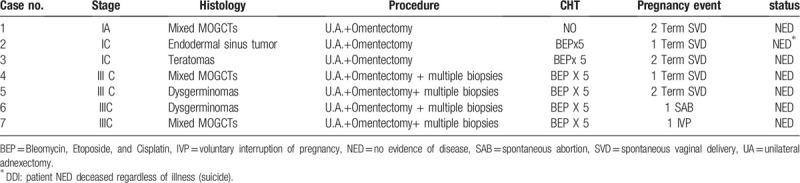
Pregnancy outcome.

### 3.1 Statistical analysis

**Figure 1:**
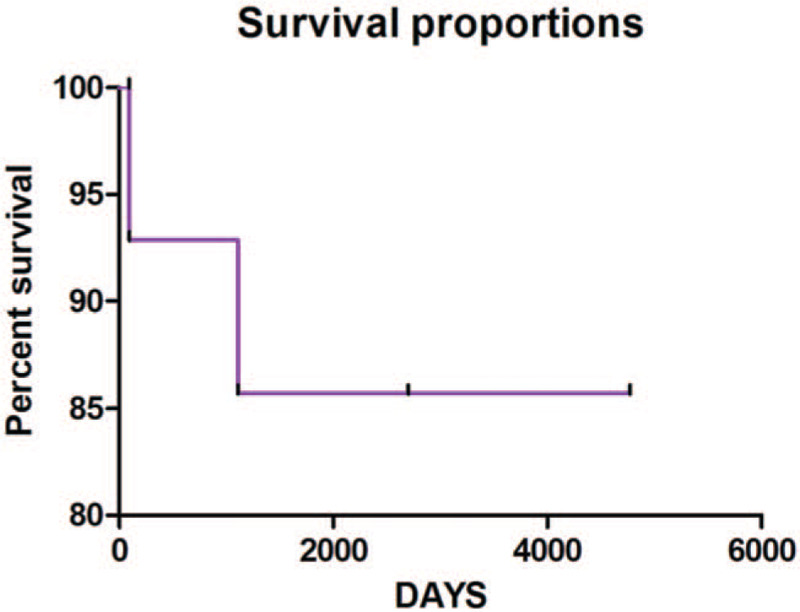
Kaplan–Meier estimates in patients with malignant ovarian germ cell tumors (MOCGTs).

In order to report the survival analysis of patients with MOCGTs, a nonparametric statistical analysis was used represented by the Kaplan–Meier estimator (Fig. 1).

## 4 Discussion

About 60% of OGCTs are diagnosed in patients under the age of 20, and one-third of these are malignant.^[16]^ Primary tumor site, histologic subtype, and metastasis could represent significant prognostic factors for survival.^[17]^ Patients in our study had demographic and clinical features similar to those reported in other series, as regards age, histologic subtype, and primary tumor site.^[18]^ Moreover, the ovaries and dysgerminomas were, respectively, the most common primary site and histologic subtype in our research, also comparable to further reports.^[19,20]^ Furthermore, in our research, we found a high survival rate in concordance with the results of additional series.^[21]^ There are some reasons to explain this, such as the early diagnosis, unilateral localization, rare metastases,^[22]^ and high chemosensitivity^[23]^ typical of these rare tumors. Moreover, the recorded recurrence rate in our patient population is low (about 12%) with a corresponding DFS of 88% and is comparable to other reports series.^[24]^ Therefore, considering the favorable prognosis of patients with MOCGT in the early stages, FSS is considered the therapeutic standard in women who want to maintain their reproductive function.^[25,26]^ Several studies report reassuring data even in advanced MOGCTs, both on OS (87.9% at 5 years) and on the absence of alterations of the menstrual cycle and reproductive function.^[27]^ In literature, the increase in AFP levels and a different histology from the dysgerminoma have been correlated to a greater incidence of recurrences. Therefore, in these cases, a more extensive surgical treatment and a greater number of chemotherapy cycles could be indicated, after individualized evaluation of the risk factors.^[28–30]^ This is also reflected in terms of OS, which in the literature is reported to be equal to 97% in cases of dysgerminoma, and 60% (*P* < .001) in nongerminomas.^[31]^ In our experience, all cases of recurrence recorded showed a histology different from dysgerminoma, and high dosage of AFP in the only case evolved in exitus for disease. Surgical treatment of advanced states must be supplemented with chemotherapy, whose effects on fertility have been widely discussed in literature. Solheim et al^[31]^ compared patients who received 3 cycles of platinum-based chemotherapy with those who received more than three cycles. In the latter group the rate of infertility was significantly higher (*P* = .040).^[32–34]^ Some authors found a reduction of primordial follicles, and stromal fibrosis following chemotherapy, with a parallel increase in gonadotropins and reduced levels of estrogen.^[35]^ These effects seem to be closely related to the type of drugs, dose, and chemotherapy duration.^[36]^ In our study, there is no evidence of any reproductive outcome reduction following multiple chemotherapy cycles. Similar data are reported in several papers that showed that PEB versus PVC chemotherapy for MOCGTs does not appear to affect reproduction or the menstrual cycle, which normalized within 6 months in 90% of cases.^[37–39]^ Similarly, we reported that 78% of patients had regular menstrual cycles during and after chemotherapy. On the contrary, 22% of patients presented amenorrhea during chemotherapy but had regular cycles 5 months after treatment. As regards fertility preservation techniques, treatment with GnRH analogues concomitant with adjuvant chemotherapy was proposed in 8 of the 18 patients, and 4 of these had spontaneous pregnancies. In 1 case, chemotherapy was performed alone. Indeed, recent studies support that chemotherapy regimens used for these neoplasms do not show particular toxicity; therefore, the association with GnRH analogues would not change the effects on fertility compared with the execution of the isolated therapy.^[40,41]^ In subjects with ovarian dysfunction at the starting stage, the association of the GnRH analogues may be indicated. Pre-treatment anti-Mullerian hormone (AMH) serum levels and the age of each patient appear to be reliable predictive factors of ovarian activity recovery after treatment.^[13,42]^ Similarly, the literature reports that AMH dosage and a pre-treatment fertility evaluation can help in the identification of patients with deficient ovarian reserve and who could benefit from a fertility preservation technique.^[43]^ In this study, there was a very small percentage of pregnancy failure: 1 had SAB and 1 had IVP; these percentages are typically reported in a general population^[44]^; therefore, it is difficult to demonstrate a relationship.^[45]^ The literature on the possible dangerous effects of cancer treatments on pregnancy did not show an increased risk of genetic or other defects in births of women previously receiving antineoplastic treatment.^[46]^ Nevertheless, in these women, considering the limited cases studied, it would be advisable to monitor the pregnancy more strictly.^[14]^ Indeed, the limitations of this study are represented by the limited number of cases, and the retrospective analysis

## 5 Conclusion

MOGCTs are rare tumors, which mainly affect patients of reproductive age.^[13]^ The increase of therapeutic rates has moved the attention of recent studies to variations in the menstrual cycle and reproductive outcome in patients after cure. Several studies report reassuring data relating to FSS treatment of MOCGTs in early stages; therefore, in these cases conservative surgery is now consolidated as a safe procedure. Moreover, for MOGCTs in advanced stages treated conservatively, the literature reports a noncompromised DFS and OS.^[7]^ In this case, personalized follow-up should be proposed in order to consider the risk factors, timing, and nature of the relapse.^[4]^ Furthermore, our study shows that FSS in advanced stages can produce good results both on reproductive outcomes and on survival. Indeed, in our group, there was only 1 case of exitus as result of recurrence. What is more, patients after FSS maintained normal ovarian function and 5 of 5 women who attempted to get pregnant succeeded spontaneously. Also, literature studies performed on a larger population report that patients with MOGCTs undergoing FSS had reassuringly high conception rates and low premature ovarian failure rates; however, in pre-treatment counseling, the risks of this approach in such a young population should be discussed.^[41]^ Nevertheless, considering the rarity of advanced stage MOGCTs (20–30%)^[22]^ and the few cases treated conservatively, the safety of FSS is accepted but not yet fully clinically supported.^[47]^ Therefore, conservative surgery for MOGCTs in advanced stages needs a greater number of cases and meta-analysis in order to obtain a higher grade of recommendation,^[27]^ but currently may represent a therapeutic option in patients available for extended follow-up and who subscribe to informed consent.

## Author contributions

MD: (Corresponding authors) has made substantial contributions to the conception of the work, approved the submitted version, and agreed both to be personally accountable for the author's own contributions and to ensure that questions related to the accuracy or integrity of any part of the work, even ones in which she was not personally involved, are appropriately investigated, resolved, and the resolution documented in the literature.

ES has made substantial contributions to design of the work, approved the submitted version, and agreed both to be personally accountable for the author's own contributions and to ensure that questions related to the accuracy or integrity of any part of the work, even ones in which she was not personally involved, are appropriately investigated, resolved, and the resolution documented in the literature.

VL has made substantial contributions to the acquisition and analysis of data, approved the submitted version, and agreed both to be personally accountable for the author's own contributions and to ensure that questions related to the accuracy or integrity of any part of the work, even ones in which author she was not personally involved, are appropriately investigated, resolved, and the resolution documented in the literature.

AP has made substantial contributions to drafting the paper and its subsequent revision. He has also approved the submitted version and agreed both to be personally accountable for the author's own contributions and to ensure that questions related to the accuracy or integrity of any part of the work, even ones in which he was not personally involved, are appropriately investigated, resolved, and the resolution documented in the literature

RL has made substantial contributions to drafting the paper and to its subsequent revision. He has also approved the submitted version and agreed both to be personally accountable for the author's own contributions and to ensure that questions related to the accuracy or integrity of any part of the work, even ones in which he was not personally involved, are appropriately investigated, resolved, and the resolution documented in the literature

CM has made substantial contributions to the acquisition and analysis of data, approved the submitted version, and agreed both to be personally accountable for the author's own contributions and to ensure that questions related to the accuracy or integrity of any part of the work, even ones in which she was not personally involved, are appropriately investigated, resolved, and the resolution documented in the literature.

AD has made substantial contributions to the design of the study, approved the submitted version, and agreed both to be personally accountable for the author's own contributions and to ensure that questions related to the accuracy or integrity of any part of the work, even ones in which she was not personally involved, are appropriately investigated, resolved, and the resolution documented in the literature.

GC has made substantial contributions to drafting the work and its subsequent revision. He has also approved the submitted version and agreed both to be personally accountable for the author's own contributions and to ensure that questions related to the accuracy or integrity of any part of the work, even ones in which he was not personally involved, are appropriately investigated, resolved, and the resolution documented in the literature.
